# Do betaine lipids replace phosphatidylcholine as fatty acid editing hubs in microalgae?

**DOI:** 10.3389/fpls.2023.1077347

**Published:** 2023-01-19

**Authors:** Danielle Yvonne Hoffmann, Yair Shachar-Hill

**Affiliations:** Department of Plant Biology, Michigan State University, East Lansing, MI, United States

**Keywords:** microalgae, lipid, acyl editing, phosphatidylcholine, triacylglyceride (TAG)

## Abstract

Acyl editing refers to a deacylation and reacylation cycle on a lipid, which allows for fatty acid desaturation and modification prior to being removed and incorporated into other pools. Acyl editing is an important determinant of glycerolipid synthesis and has been well-characterized in land plants, thus this review begins with an overview of acyl editing in plants. Much less is known about acyl editing in algae, including the extent to which acyl editing impacts lipid synthesis and on which lipid substrate(s) it occurs. This review compares what is known about acyl editing on its major hub phosphatidylcholine (PC) in land plants with the evidence for acyl editing of betaine lipids such as diacylglyceryltrimethylhomoserine (DGTS), the structural analog that replaces PC in several species of microalgae. In land plants, PC is also known to be a major source of fatty acids and diacylglycerol (DAG) for synthesis of the neutral lipid triacylglycerol (TAG). We review the evidence that DGTS contributes substantially to TAG accumulation in algae as a source of fatty acids, but not as a precursor to DAG. We conclude with evidence of acyl editing on other membrane lipid substrates in plants and algae apart from PC or DGTS, and discuss future analyses to elucidate the role of DGTS and other betaine lipids in acyl editing in microalgae.

## Introduction

Research in algal lipid metabolism has undergone a resurgence in recent years (Merchant et al., 2012; Du and Benning, 2016; Goncalves et al., 2016), particularly due to interest in the neutral lipid triacylglycerol (TAG), which is a source of nutritionally valuable fatty acids and a key feedstock for biodiesel fuel production. Both the quantity and composition of TAG are important and are governed by fluxes through intermediate glycerolipids pools such as membrane lipids (Goncalves et al., 2013; Légeret et al., 2016; Young and Shachar-Hill, 2021). The process of acyl editing, which is used to mean the addition and removal of an acyl group on a lipid with the potential for tailoring and modification and subsequent incorporation into another lipid pool, plays a central role in fluxes between membrane lipids and TAG. Much of what is currently known about acyl editing has been determined in land plants, which utilize the extraplastidial lipid phosphatidylcholine (PC) as the main hub of acyl editing and fatty acid modification, and which also contributes to TAG synthesis. However, a range of algae lack PC and rather contain ether-linked betaine lipids (Kato et al., 1996; Künzler and Eichenberger, 1997), thus raising the question: To what extent do betaine lipids replace the functions of PC in algae? A better understanding of acyl editing is crucial to efforts in algal lipid engineering; improving the yield and composition of algal TAG without this knowledge is likely to remain a hit-or-miss affair.

**Figure 1:**
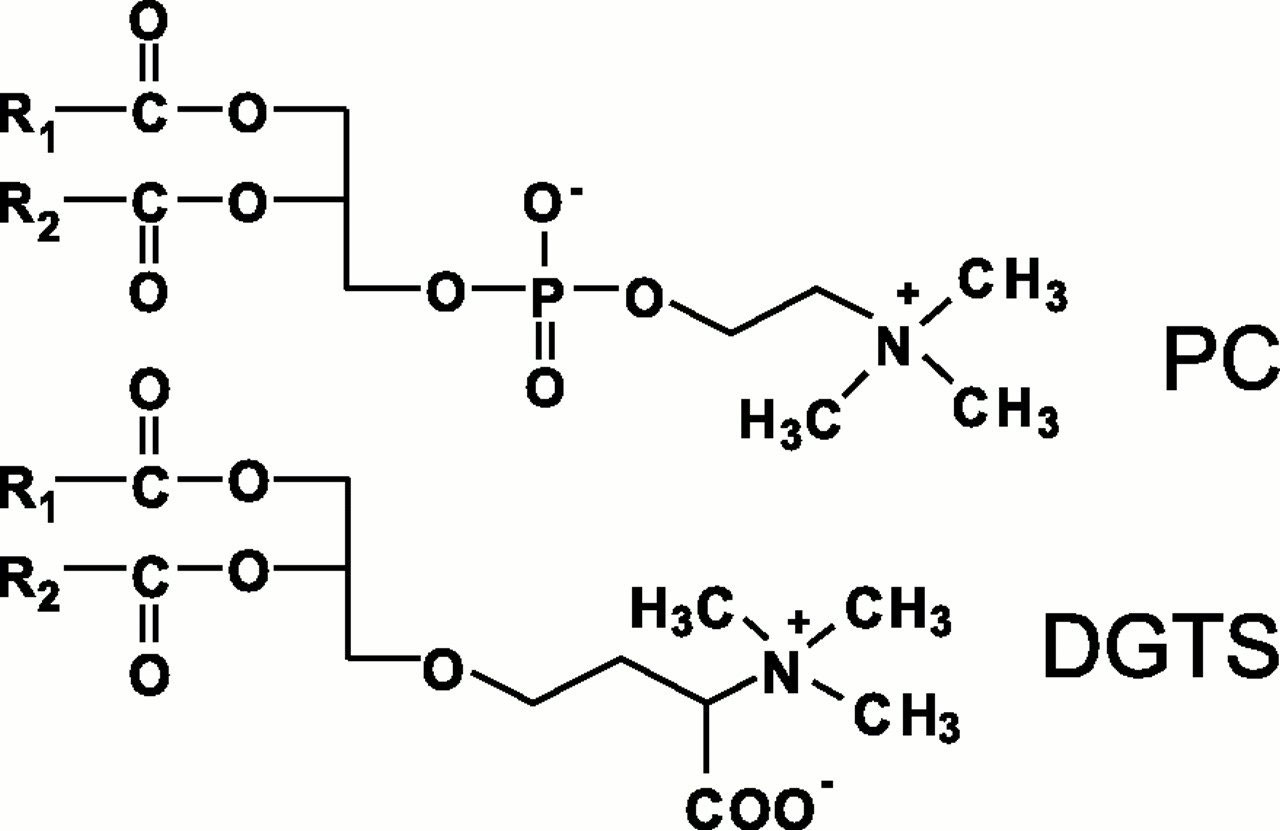
Structures of PC and DGTS. Figure reproduced with permission from Klug and Benning, 2001. Copyright (2001) National Academy of Sciences, U.S.A. DGTS, diacylglyceryl-N,N,N-trimethylhomoserine; PC, phosphatidylcholine.

PC serves as the major acyl hub in plants, where it is the predominant lipid outside of the chloroplast in most plant cells. PC plays a central role in several aspects of plant lipid metabolism by acting as the major substrate for extraplastidial acyl modifications, in lipid trafficking between subcellular compartments, and in conducting fluxes toward TAG synthesis (Miquel and Browse, 1992; Bates et al., 2012; Chen et al., 2015). In many microalgal species, betaine lipids are present in inverse proportion to PC levels, replacing it completely in some cases and partially in others. Due to this inverse relationship and the structural similarity of betaine lipids and PC (**Figure 1**), it has been hypothesized that betaine lipids play analogous roles to PC in algae. The betaine lipid diacylglyceryl-N,N,N-trimethylhomoserine (DGTS) is widely distributed in nonflowering groups such as green algae, lichens, mosses, and ferns and is absent in seed plants and flowering plants (Künzler and Eichenberger, 1997), suggesting that the capacity to synthesize DGTS was lost in the spermatophyte ancestor to seed plants. Some bacteria are capable of synthesizing DGTS in response to phosphate starvation (Geiger et al., 1999), and DGTS is present in some fungi as well (Künzler and Eichenberger, 1997). It is believed that a green algae ancestor was capable of producing DGTS, thus creating a “DGTS branch.” However, some algae in this branch lack DGTS, indicating that some organisms either reduced or lost the capacity for betaine lipid synthesis. The replacement of PC with DGTS may have evolved as a stress acclimation response to low phosphorus or low temperature environments.

In this review, we compare the roles of PC in plant lipid metabolism with what is known about the role of betaine lipids, particularly DGTS, in algae. The first sections below describe the metabolic roles of PC in membrane synthesis and oil accumulation in plants. The subsequent sections present evidence in favor and against DGTS replacing the different functions of PC in algae and discuss alternative acyl editing substrates apart from PC or DGTS. The evidence outlined for PC’s role in plants serves as a pointer to methods that have or can be used in the future to address these questions in algae. Rational engineering of algal lipid metabolism such as TAG accumulation and composition will require knowledge of their acyl hubs, as evidenced by prior attempts to engineer the accumulation of particular fatty acids in oilseeds, in which flux through PC represented a major bottleneck (Bates and Browse, 2011).

## Overview of acyl editing in plants

**Figure 2:**
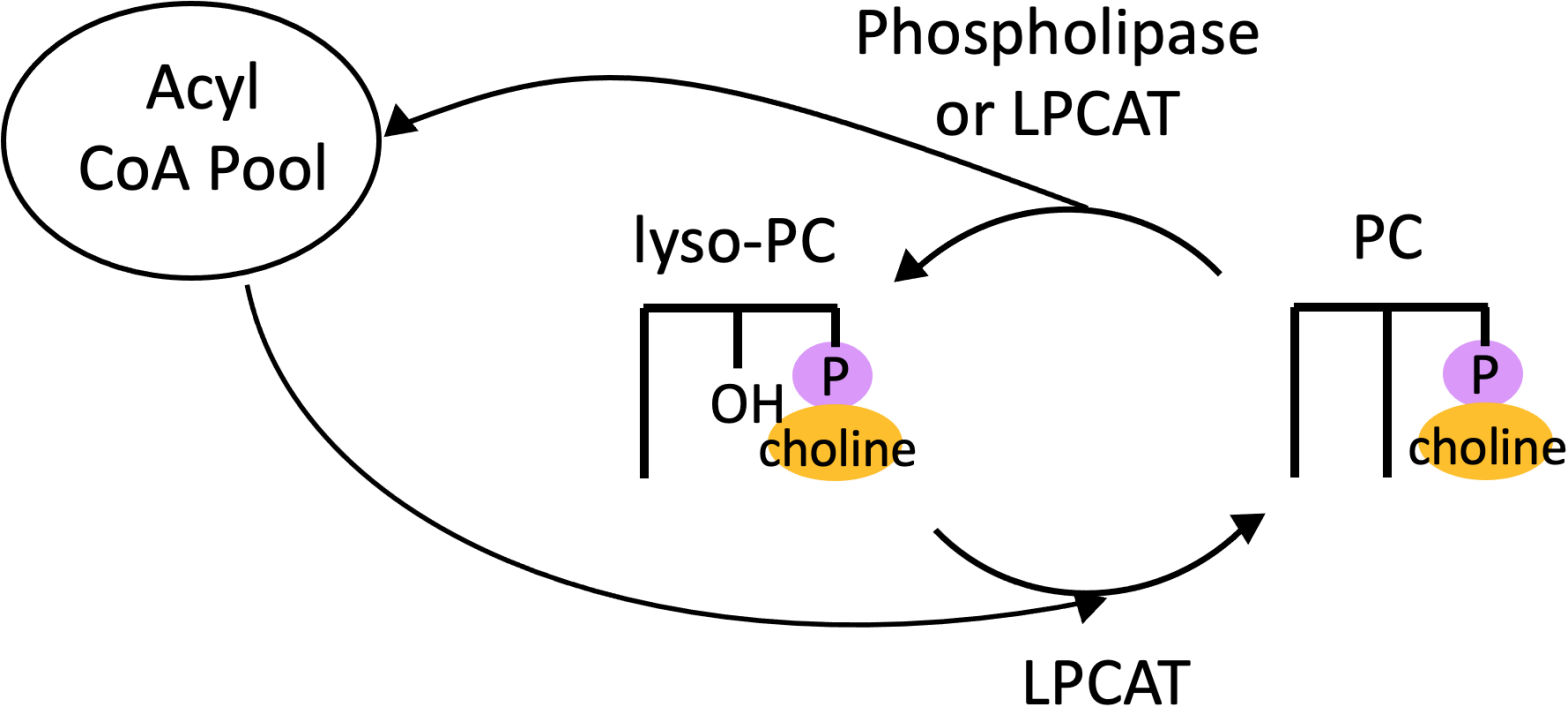
The Lands Cycle. Cycle of deacylation of PC *via* either a phospholipase or LPCAT to produce lyso-PC, which is then reacylated by LPCAT to regenerate PC. LPCAT, acyl-CoA:lysophosphatidylcholine acyltransferase; PC, phosphatidylcholine.

The process of acyl editing is an important determinant of eukaryotic membrane lipid synthesis as well as TAG accumulation and has been well-characterized in land plants. Therefore, we begin this review with an overview of acyl editing and related functions in plants. The existence of a deacylation and reacylation cycle on PC, the most abundant membrane lipid in animals and the major extraplastidic one in plants, was originally inferred from ^14^C-tracer experiments. Supplying rat lung tissue with ^14^C-acetate and ^14^C-glycerol resulted in a ratio of radioactivity in fatty acids compared to glycerol that was much higher in phospholipids than in TAG, implying that turnover of fatty acids occurred on PC (Lands, 1958). It was hypothesized that this occurred *via* the action of a phospholipase generating lysophosphatidylcholine (lyso-PC, PC with one fatty acid removed), which was then reacylated to form PC (Lands, 1960). In microsomes of developing safflower cotyledons, ^14^C-labeling revealed rapid exchange of fatty acids between diacylglycerol (DAG) and PC (Stobart and Stymne, 1985), and evidence was found that this acyl exchange was catalyzed by the forward and reverse reactions of acyl-CoA:lysophosphatidylcholine acyltransferase (LPCAT) (Stymne and Stobart, 1984). Thus, acyl exchange on PC, termed “the Lands’ cycle,” could proceed *via* two possible mechanisms: the action of a phospholipase followed by reacylation by LPCAT, or by both forward and reverse reactions catalyzed by LPCAT (**Figure 2**).

A study utilizing ^14^C-acetate and ^14^C-glycerol labeling in pea leaves found that the majority of newly made fatty acids were rapidly incorporated into PC rather than proceeding *via* the *de novo* synthesis pathway (Bates et al., 2007). Here, “*de novo* synthesis” refers to the Kennedy pathway in which glycerol-3-phosphate (G3P) is acylated to form lyso-phosphatidic acid, which is then acylated to form phosphatidic acid (PA), whose phosphate group is removed to form DAG. PC is then synthesized from DAG *via* cytidine-5′-diphosphocholine:diacylglycerol cholinephosphotransferase (CPT). This labeling study in pea leaves found that over 90% of ^14^C-labeled PC molecules contained one ^14^C-labeled fatty acid and one unlabeled fatty acid, and 62% of the label was found at the *sn*-2 position and 38% at the *sn*-1 position (Bates et al., 2007). PC acyl editing was found to be the major route of newly made fatty acid flux in plant leaves (Bates et al., 2007), a process that allows polyunsaturated fatty acids (PUFAs) to be produced on PC and then distributed to other lipids.

Labeling with ^14^C-acetate and ^14^C-glycerol during TAG synthesis in developing soybean embryos also revealed the direct incorporation of newly made fatty acids into PC similar to that observed in pea leaves (Bates et al., 2009). In addition, 86% of newly synthesized fatty acids in PC were found at the *sn*-2 position, suggesting that acyl editing occurs primarily at this location (Bates et al., 2009). It was concluded that the major flux of newly made fatty acids proceeded through PC *via* acyl editing in this oilseed, highlighting the importance of acyl editing in lipid synthesis in TAG accumulation as well as membrane synthesis.

## Phosphatidylcholine serves as the substrate of fatty acid modification and key intermediate in TAG synthesis in plants

**Figure 3:**
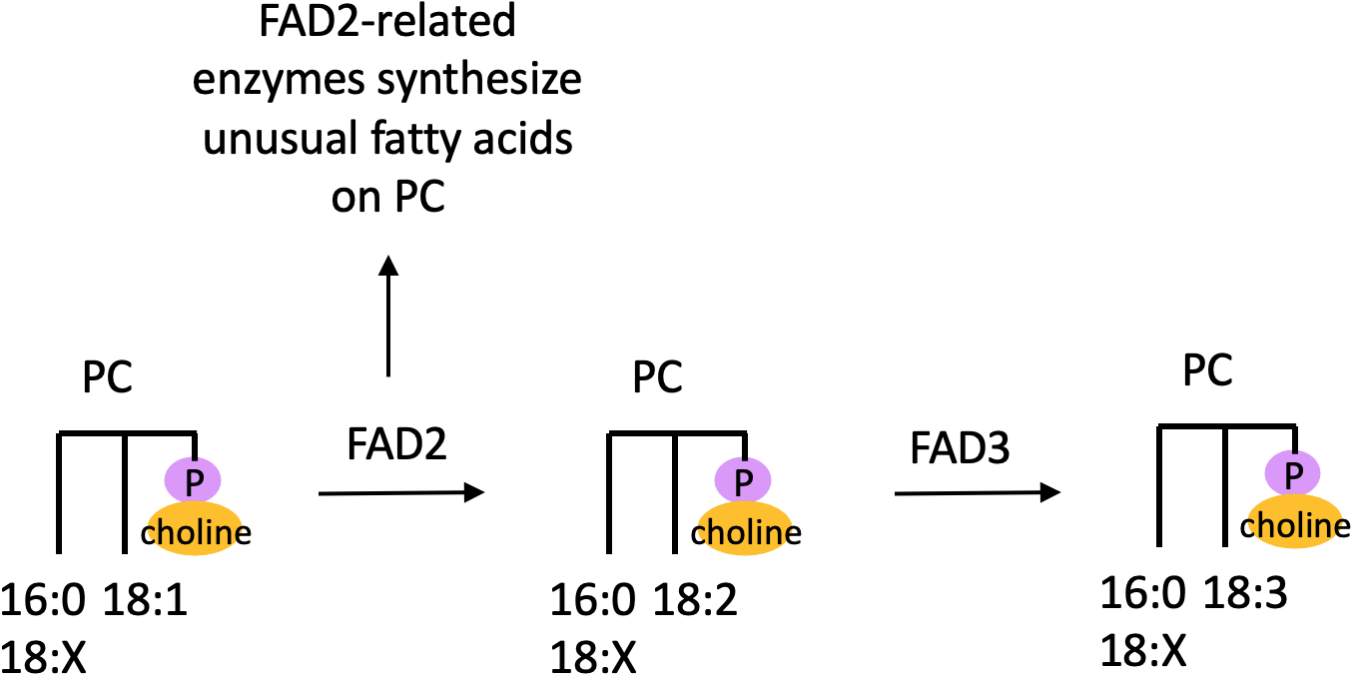
Desaturation and production of unusual fatty acids on PC. Desaturation and unusual fatty acid synthesis occurs on the *sn-2* position of PC. Here ‘18:X’ refers to a fatty acid with 18 carbons and an unspecified number of double bonds. FAD, Fatty Acid Desaturase; PC, phosphatidylcholine.

In plants, PC is the major site of extraplastidial desaturation to produce PUFAs, as studies have demonstrated that oleic acid (18:1Δ9) is desaturated to form linoleic acid (18:2Δ9,12) in a lipid-linked manner on PC (Sperling and Heinz, 1993; Sperling et al., 1993; **Figure 3**). This work found that desaturation could occur at the *sn-1* or *sn-2* position of PC based on extraction of PC from *in vivo* cell cultures followed by treatment with a stereospecific lipase (Sperling et al., 1993), but given that in PC polyunsaturated C18-fatty acids are more abundant at the *sn-2* position, this is the major site of desaturation on PC *in planta*. Desaturation of 18:1 to 18:2 on PC is catalyzed by the desaturase Fatty Acid Desaturase 2 (FAD2), while desaturation of 18:2 to 18:3 on PC is catalyzed by FAD3 (**Figure 3**). These desaturase genes were discovered by isolating *Arabidopsis* mutants deficient in the fatty acid resulting from their activity (Miquel and Browse, 1992; Browse et al., 1993), and these genes were cloned and demonstrated to complement the mutant phenotype (Arondel et al., 1992; Okuley et al., 1994). Thus, it is believed that PC is synthesized from 16:0/18:1 or 18:1/18:1 DAG, with 16:0 primarily at the *sn*-1 position while 18:1 can occur at both the *sn*-1 and *sn*-2 positions, after which 18:1 is desaturated to form 18:2 followed by 18:3 (**Figure 3**; Browse and Somerville, 1991). Given that there is a lack of evidence for DAG or other lipids in the endoplasmic reticulum (ER) serving as substrates for desaturation, PC is believed to be the major site of fatty acid desaturation in this membrane system.

**Figure 4:**
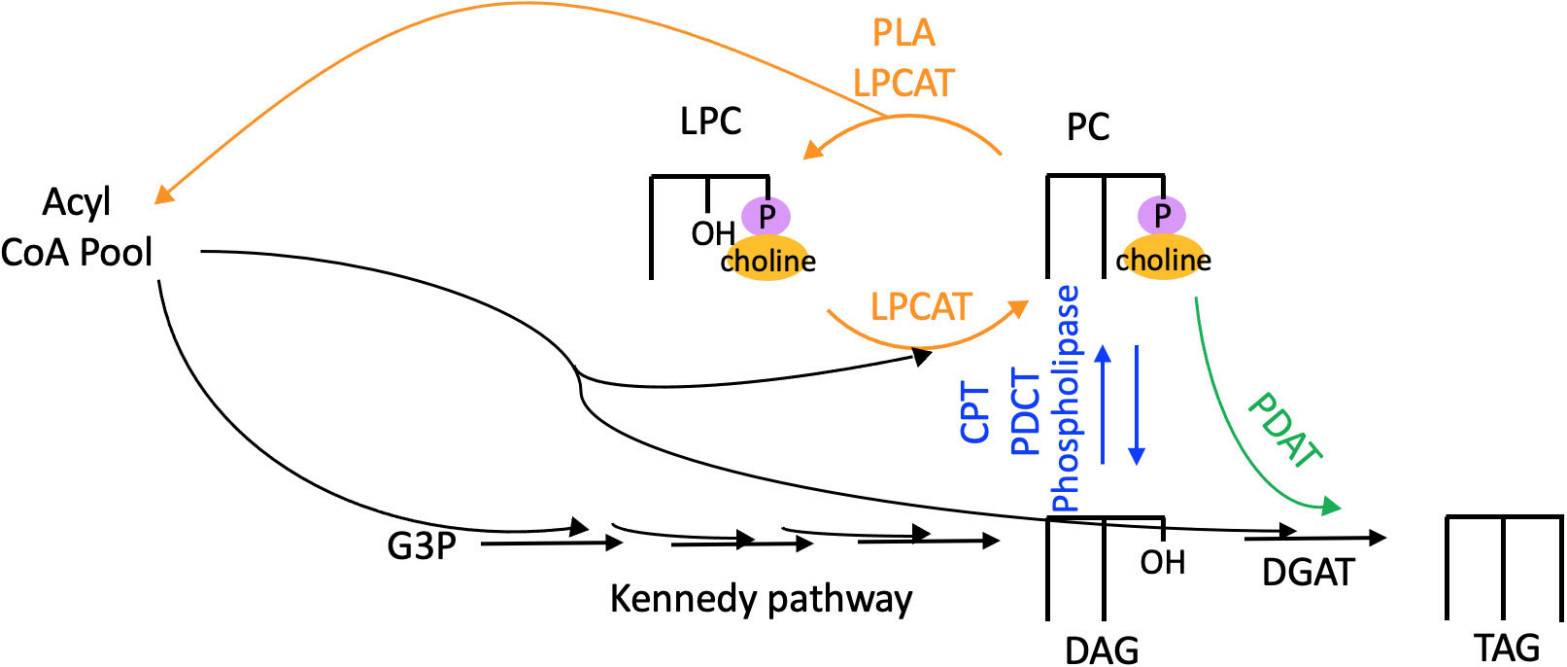
Routes of acyl flux through PC into TAG. Orange arrows, PC acyl editing reactions. Blue arrows, headgroup exchange for PC-DAG conversion. Green arrows, transfer of an acyl group from PC onto TAG *via* the acyltransferase PDAT. CPT, cytidine-5′-diphosphocholine:diacylglycerol cholinephosphotransferase; DAG, diacylglycerol; DGAT, diacylglycerol acyltransferase; G3P, glycerol 3-phosphate; LPC, lyso-phosphatidylcholine; LPCAT, acyl-CoA:lysophosphatidylcholine acyltransferase; PC, phosphatidylcholine; PDAT, phospholipid:diacylglycerol acyltransferase; PDCT, phosphatidylcholine:diacylglycerol cholinephosphotransferase; PLA, phospholipase; TAG, triacylglycerol.

Thus, PC is the site of extraplastidial desaturation, and acyl flux through PC is a major route for PUFA incorporation into TAG. The fatty acids of PC can reach TAG by three major routes as shown in **Figure 4**: 1) Fatty acids may be removed from PC by a phospholipase or acyltransferase and enter the acyl-CoA pool, where they are then available for glycerolipid synthesis. 2) PC may be converted into DAG, the precursor for TAG synthesis, by the reverse action of cytidine-5′-diphosphocholine:diacylglycerol cholinephosphotransferase (CPT), by phosphatidylcholine:diacylglycerol cholinephosphotransferase (PDCT), or by the action of phospholipase C or phospholipase D followed by phosphatidic acid phosphatase to remove the phosphate headgroup. 3) A fatty acid may be directly transferred from PC onto the *sn*-3 position of DAG to produce TAG by the action of phospholipid:diacylglycerol acyltransferase (PDAT) (Dahlqvist et al., 2000; Stahl et al., 2004). Each of these routes of acyl flux from PC can contribute to increase the PUFA content in TAG.

In addition to desaturation, PC also acts as the substrate for several unusual fatty acid modifications (**Figure 3**). Mutations in plant fatty acid desaturases can give rise to alternate enzymatic activities such as hydroxylation, epoxygenation, and triple-bond formation. For example, many variants of the FAD2 desaturase, which desaturates oleic acid (18:1) to linoleic acid (18:2) on PC, have been discovered. In castor bean, a hydroxylase acts on the Δ12 position of oleic acid on the *sn-2* position of PC to produce ricinoleic acid, and this hydroxylase is a homolog of FAD2 (Bafor et al., 1991; Van De Loo et al., 1995). An acetylenase in *Crepis alpina* is a variant of FAD2 that introduces a triple bond at the Δ12 position of linoleic acid on PC to form crepenynic acid, and this unusual fatty acid then accumulates in TAG (Banas et al., 1997). In addition, a Δ12-epoxygenase from *Crepis palaestina* catalyzes the formation of vernolic acid from linoleic acid, presumably on PC (Lee et al., 1998). Thus, PC is the key substrate for synthesizing PUFAs as well as uncommon fatty acids.

Although PC is the site of synthesis of unusual fatty acids, some plants contain high amounts of unusual fatty acids in TAG while PC contains low levels of these fatty acids. For example, in developing endosperms of castor bean, the hydroxylated fatty acid ricinoleic acid accumulates to only 5% in PC while it accumulates to ~85% in TAG despite its synthesis on PC (Stahl et al., 1995). Thus, acyl flux through the *sn-2* position of PC must occur with high efficiency and selectivity. However, when the castor bean hydroxylase is transgenically expressed in *Arabidopsis*, hydroxy-fatty acids only accumulate to ~17% in seed TAG (Broun and Somerville, 1997). Isotopic labeling analyses using ^14^C-glycerol revealed a ~50% reduction in label incorporation in total lipids, primarily due to a reduction in the use of *de novo* DAG for PC synthesis (Bates and Browse, 2011). Thus, flux through PC can represent a bottleneck for the accumulation of particular fatty acids in TAG (Bates and Browse, 2011).

Another example of PC acting as a control point for fluxes of unusual fatty acids is the unusual fatty acid petroselinic acid (18:lcisΔ6), which is present in low levels in PC (15-20%) and high levels in TAG (70-75%) in both carrot and coriander seed endosperm (Cahoon and Ohlrogge, 1994). However, ^14^C-acetate time course labeling experiments revealed that at early time points, radiolabel was the most concentrated in PC and entered it at the highest rates, while at later time points radiolabel accumulated most strongly in TAG, and primarily (80-85%) in petroselinic acid (Cahoon and Ohlrogge, 1994). These results suggest that there is significant flux of petroselinic acid from PC into TAG. Similar results were found in *Thunbergia alata*, whose unusual monoenoic fatty acid 16:1Δ6 comprises >80% of TAG fatty acids and which appears first in PC (Schultz and Ohlrogge, 2000). Therefore, in order to engineer high levels of select fatty acids in transgenic oilseeds or algae, the major fluxes of TAG synthesis, including the sites of modification and distribution, must be elucidated.

## Mechanisms of acyl editing and acyl flux through PC into TAG

**Figure 5:**
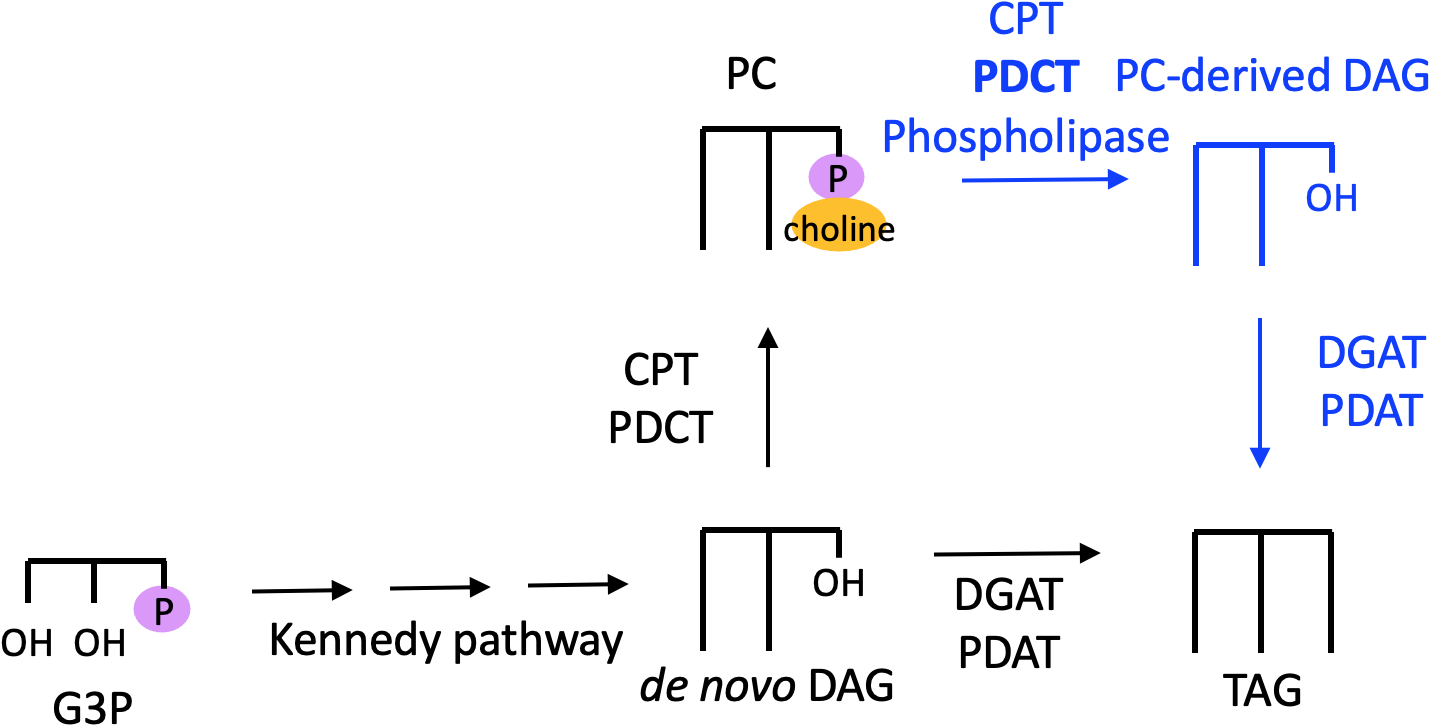
PC-derived DAG pathway to TAG synthesis. Black text and arrows represent the *de novo* synthesis pathway to PC and TAG, blue text and arrows represent the PC-derived DAG pathway to produce TAG. The enzyme PDCT is shown in bold because it is believed to catalyze the major PC-DAG conversion. CPT, cytidine-5′-diphosphocholine:diacylglycerol cholinephosphotransferase; DAG, diacylglycerol; DGAT, diacylglycerol acyltransferase; G3P, glycerol 3-phosphate; PC, phosphatidylcholine; PDAT, phospholipid:diacylglycerol acyltransferase; PDCT, phosphatidylcholine:diacylglycerol cholinephosphotransferase; TAG, triacylglycerol.

PC conversion into DAG for TAG synthesis has been shown to be an important biochemical pathway in oilseeds. For instance, ^14^C-acetate and ^14^C-glycerol labeling of developing soybean embryos demonstrated that over 95% of TAG synthesis was found to be derived from DAG generated from PC, or “PC-derived DAG” (Bates et al., 2009; **Figure 5**). This TAG synthesis pathway was also found to be significant in *Arabidopsis* seeds, with ^14^C-glycerol labeling revealing that PC-derived DAG is utilized for as much as 93% of TAG synthesis (Bates and Browse, 2011). Thus, in several oilseeds *de novo*-synthesized DAG is converted to PC, which is converted back to DAG, which produces TAG (**Figure 5**). However, developing castor-bean endosperm appears to utilize *de novo*-synthesized DAG to produce TAG (Bafor et al., 1991), so this pathway is not ubiquitously utilized by all plants.

PC-derived DAG can be synthesized by the reverse action of CPT, which produces PC (Slack et al., 1983). PC-DAG interconversion can also be catalyzed by PDCT, which reversibly exchanges the phosphocholine headgroup between PC and DAG. DAG may also be produced from PC *via* the action of phospholipase C, or *via* phospholipase D followed by phosphatidic acid phosphatase. However, characterization of the *Arabidopsis* rod1 mutant, which encodes PDCT, revealed that at least 40% of the PUFAs in TAG are derived from PC-derived DAG produced by this enzyme (Lu et al., 2009). Thus, PDCT catalyzes the major PC-DAG interconversion in developing *Arabidopsis* seeds (**Figure 5**).

In addition to headgroup removal, PC can also contribute to TAG synthesis *via* the well-known PDAT enzyme, which has been characterized in yeast, oilseeds, and other plant tissues (Dahlqvist et al., 2000; Stahl et al., 2004). PDAT catalyzes an acyl-CoA independent pathway of TAG formation by transferring an acyl group from the *sn*-2 position of PC onto DAG (**Figure 4**). PDAT is known to conduct significant flux into TAG in yeast and oilseeds, although knockout of *PDAT* in Arabidopsis resulted in no statistically significant difference in seed lipid content, likely due to compensation by diacylglycerol acyltransferase (DGAT) activity (Mhaske et al., 2005). In microalgae, *pdat* insertional mutants appeared to accumulate 25% less TAG during nitrogen deprivation compared to the parental strain (Boyle et al., 2012), suggesting that it may be a determinant of TAG accumulation in algae. However, artificial miRNA-silenced PDAT knockdowns in *Chlamydomonas reinhardtii* did not show significant reduction of TAG under N-deprivation (Yoon et al., 2012), and further characterization of *C. reinhardtii pdat* knockout mutants suggested that PDAT primarily regulates TAG biosynthesis under favorable conditions rather than persistent stress conditions (Lee et al., 2022). Thus, PDAT may play a diminished role in TAG synthesis in algae compared to yeast and plants.

Two studies probed the enzymes underlying the acyl editing mechanism in *Arabidopsis* seeds by generating double mutants in both LPCAT genes of *Arabidopsis* (*lpcat1/lpcat2*) (Bates et al., 2012; Wang et al., 2012), which deacylate and reacylate lyso-PC and PC in the acyl editing cycle (**Figure 2**). Both studies observed a reduction of PUFA content in seeds by ~10% in the *lpcat1/lpcat2* double mutant (Bates et al., 2012; Wang et al., 2012). In addition, both studies used ^14^C-labeling to confirm that in the double mutants, nascent fatty acids were incorporated in the *de novo* synthesis route of DAG followed by PC rather than by direct incorporation into PC characteristic of acyl editing (Bates et al., 2012; Wang et al., 2012). Both studies also found that the *lpcat1/lpcat2* double mutants accumulated lyso-PC, indicating they were impaired in their ability to reacylate lyso-PC to PC. Thus, these studies provided strong evidence that the LPCAT genes are crucial components of the acyl editing cycle in *Arabidopsis*. In addition, Bates et al. generated a triple mutant *lpcat1/lpcat2/rod1* that was also deficient in PDCT, which interconverts DAG and PC. The triple mutant’s PUFA content in seed TAG was reduced by about two-thirds (Bates et al., 2012), suggesting that PDCT and the LPCAT genes are responsible for the majority of fatty acid flux in and out of PC, *via* PC-DAG interconversion by PDCT and acyl editing by LPCAT.

Early evidence in microsomes of developing safflower cotyledons suggested that acyl exchange on PC was catalyzed by the forward and reverse reactions of LPCAT (Stymne and Stobart, 1984), and this was later confirmed by expressing *Arabidopsis* LPCAT2 in yeast and measuring LPCAT activity by the rate of incorporation of ^14^C-18:1 fatty acid into PC (Lager et al., 2013). These assays revealed that LPCAT1 and LPCAT2 can catalyze both the acylation and deacylation of PC (Lager et al., 2013). While both LPCATs can acylate and deacylate at the *sn-1* position, the *sn*-2 position is strongly preferred (Lager et al., 2013). Both LPCAT enzymes showed low activity toward 16:0 fatty acid, and thus are probably not involved in acylating or deacylating 16:0 on PC (Lager et al., 2013). Thus, LPCAT is reversible *in vitro*, although it is still possible that acyl editing could proceed *via* the action of a phospholipase followed by the forward reaction of LPCAT to regenerate PC.

**Figure 6:**
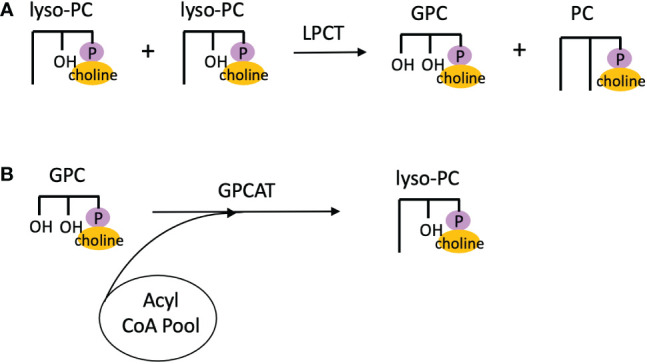
Additional PC acyl editing reactions. **(A)** Lyso-PC:lyso-PC transacylation *via* LPCT activity. **(B)** Acylation of GPC by GPCAT activity. GPC, glycerophosphocholine; GPCAT, acyl-CoA:glycerophosphocholine acyltransferase; LPCT, lysphosphatidylcholine transacylase; PC, phosphatidylcholine.

In addition to LPCAT reversibility, Lager et al. used microsomes of developing safflower seeds to characterize other reactions involved in PC acyl editing. By performing isotopic labeling experiments with [^14^C]18:1-lyso-PC and [^14^C]choline, they demonstrated that lyso-PC:lyso-PC transacylation occurred, and named this activity lysphosphatidylcholine transacylase (LPCT) (Lager et al., 2015). Thus, LPCT activity utilizes two lyso-PC molecules and produces PC and glycerophosphocholine (GPC; **Figure 6A**). By incubating microsomal preparations of various developing oilseeds in [^14^C]GPC and 18:1-CoA and measuring the radioactive PC product, acyl-CoA:glycerophosphocholine acyltransferase (GPCAT) activity was also demonstrated (Lager et al., 2015), which catalyzes the acylation of GPC to lyso-PC (**Figure 6B**). The enzyme responsible for LPCT activity has yet to be identified, but the GPCAT gene has been identified and cloned in *Arabidopsis*, and GPCAT homologs have been found across other eukaryotic clades including green algae (Głąb et al., 2016).

Many studies on developing seeds are performed *in vitro*, and one study sought to compare *in vitro* versus *in vivo* growth on acyl lipid metabolism in *Camelina sativa* leaves (Klińska et al., 2021). Significant differences were observed between the two growth conditions, with *in vivo* leaves containing over twice the total fatty acid content of *in vitro* leaves, *in vivo* conditions resulting in a higher degree of desaturation in fatty acids compared to *in vitro*, and the dominant lipid in *in vitro* conditions being PC rather than monogalactosyldiacylglycerol (MGDG) (Klińska et al., 2021). Interestingly, higher activity of acyl-CoA:lysophospholipid acyltransferases (LPLATs) was observed *in vitro*, as well as an increased rate of phospholipid remodeling (Klińska et al., 2021). Collectively, these results indicate that acyl lipid metabolism and acyl editing differ substantially between *in vitro* and *in vivo* conditions, and results from one condition should not be extrapolated to the other.

Acyl editing is also known to occur in response to stress, such as temperature stress, salt stress, and nutrient deprivation. For instance, lipidomic profiling during chilling stress in barley roots revealed that as particular molecular species of PC decreased, those molecular species increased in PA (Vilchez et al., 2021). Transcriptional analysis of genes involved in acyl editing revealed that the barley LPCAT gene was upregulated within five hours of cold recovery, and phospholipase D activity rose during cold treatment and after five hours of cold recovery (Vilchez et al., 2021). Thus, it is likely that PC is hydrolyzed to produce PA during cold stress and recovery, and that acyl editing of PC plays an important role in chilling stress and recovery.

## Betaine lipids may replace PC as a fatty acid editing hub in algae

In flowering plants, PC is the major extraplastidial membrane lipid, but some algae and nonflowering plants contain DGTS and/or other betaine lipids in place of PC as the major extraplastidial lipid. DGTS is a betaine lipid that is structurally highly similar to PC, but it lacks phosphate in its headgroup and rather contains an ether bond linking the headgroup to the glycerol backbone (**Figure 1**). Due to its structural similarity to PC, DGTS is widely believed to replace the function of PC in extrachloroplastic membranes, and several studies in algae and diatoms containing DGTS have found evidence that DGTS may substitute for several of the functions of PC.

The green microalga *C. reinhardtii* is the most studied alga in lipid and other areas of metabolism. *C. reinhardtii* lacks PC and rather contains DGTS, and an inverse relationship between the quantities of these two lipids occurs in many species of microalgae (Dembitsky, 1996). When ^14^C-oleic acid is supplied exogenously to *C. reinhardtii* cells, label is rapidly incorporated into DGTS and it contains the majority of the radiolabel, similar to that which is observed in PC in flowering plants (Schlapfer and Eichenberger, 1983; Giroud and Eichenberger, 1989). The golden-brown microalga *Ochromonas danica* contains the betaine lipids DGTS and diacylglycerylhydroxymethyltrimethyl-β-alanine (DGTA), and small amounts of PC. When this alga was incubated with ^14^C-oleic acid, the majority of the radiolabel was incorporated into DGTS, suggesting that DGTS is the primary acceptor of exogenous oleic acid (Vogel and Eichenberger, 1992). Similar findings were observed in the brown algae *Fucus vesiculosus* and *Ascophyllum nodosum*, which contain DGTA and minor quantities of PC. In these algae, labeling with ^14^C-acetate revealed DGTA to be the lipid with the highest incorporation of radioactivity (Jones and Harwood, 1993). Thus, in algae containing ether-linked betaine lipids in place of PC, supplying exogenous labeled fatty acids or acetate results in the early and strong incorporation of label into betaine lipid, analogous to the rapid incorporation of radiolabel into PC observed in land plants.

In addition to being the first major lipid into which exogenous radiolabel is incorporated, DGTS also appears to be the site of extraplastidial desaturation of C18 fatty acids akin to PC. When ^14^C-oleic acid was supplied exogenously to *C. reinhardtii*, radiolabel first appeared in molecular species of DGTS containing 18:1, and then shifted to species containing 18:2, followed by species containing 18:3Δ5,9,12, suggesting that C18 fatty acid desaturation occurs on DGTS (Schlapfer and Eichenberger, 1983; Giroud and Eichenberger, 1989). Similar results were obtained in a pulse-chase labeling experiment in the golden-brown microalga *O. danica*, in which labeling with ^14^C-oleic acid resulted in radiolabel being primarily concentrated in DGTS in its 18:1 and 18:2 fatty acids (Vogel and Eichenberger, 1992). During the chase, radiolabel decreased very rapidly in 18:1 while it decreased more slowly in 18:2 fatty acids, and radiolabel increased strongly in 18:3 and 18:4 fatty acids (Vogel and Eichenberger, 1992). This suggested that 18:1 is desaturated on DGTS to produce 18:3 and 18:4 fatty acids. Thus, in algae containing DGTS rather than PC, DGTS appears to take on PC’s role as a substrate of extraplastidial fatty acid desaturation.

Labeling experiments in oilseed plants using ^14^C-acetate have demonstrated that radiolabeled fatty acids are rapidly incorporated into PC prior to their incorporation into DAG and TAG (Bates et al., 2009). Pulse-chase experiments in *C. reinhardtii* cells supplied with ^14^C-radiolabeled fatty acids in nutrient replete medium and then transferred into unlabeled, nitrogen-deprived medium to induce TAG accumulation revealed that radiolabel was rapidly lost from DGTS while it increased in TAG during the chase period (Xu et al., 2016). Similarly, when the brown alga *F. vesiculosus* was incubated with ^14^C-acetate, radiolabel was initially most concentrated in the betaine lipid DGTA, and over time decreased in DGTA while increasing strongly in neutral lipids, primarily TAG (Jones and Harwood, 1993). Thus, pulse-chase experiments with radiolabeled substrates indicate that fatty acids pass through betaine lipids into TAG in algae in a similar manner as PC in land plants.

In addition to pulse-chase experiments, several lipidomics-based analyses point to fatty acid flux through betaine lipids into TAG. One study utilizing subcellular lipidomics to investigate the origins of TAG formation in nitrogen-deprived *C. reinhardtii* indicated that the membrane lipids MGDG, digalactosyldiacylglycerol (DGDG), and DGTS all contribute acyl chains to TAG accumulation (Yang et al., 2018). In particular, they noted that as certain molecular species of DGTS accumulated, there was a corresponding increase in the levels of those acyl chains in TAG (Yang et al., 2018). In support of this finding, a glycerolipidomics study of the BAFJ5 starchless mutant in *C. reinhardtii* subjected to high light and nitrogen deprivation concluded that 18:3Δ9,12,15/16:0 from DGDG and 16:0/18:3Δ5,9,12 from DGTS were major contributors to 18:3 accumulation in TAG (Yang et al., 2020). The diatom *Phaeodactylum* strain Pt4 (UTEX 646) contains DGTA as the major extraplastidial lipid and minor amounts of PC (Abida et al., 2015), and analysis of its lipid molecular species composition during nitrogen deprivation indicated that the betaine lipid was the largest membrane lipid contributor of fatty acids to TAG accumulation (Popko et al., 2016). The major TAG species that accumulated were matched to a reduction in those corresponding species in MGDG and the betaine lipid, but only the decrease in the latter was large enough to account for the increase observed in TAG (Popko et al., 2016). Thus, several lines of evidence indicate that betaine lipids serve as a major source of fatty acids for TAG synthesis.

The relationship between DGTS composition and levels and TAG accumulation was further evident in knockdown transformants in the betaine lipid synthase gene (BTA1) in *C. reinhardtii*. Artificial microRNA knockdown of the BTA1 gene expression level led to a significant decrease in DGTS and MGDG contents while TAG increased 2-3 fold (Lee et al., 2017). The authors postulate that the observed decrease in MGDG was due to the indirect effect of ER stress due to the substantial decrease of DGTS. Their results indicated that the synthesis of DGTS was inhibited by the reduced expression of BTA1, thus resulting in an increased amount of DAG that could not be converted into DGTS, which was then available for TAG synthesis (Lee et al., 2017). This was evidenced by a 40% reduction in DGTS content in the BTA1 gene knockdown lines compared to wild type and empty vector controls, and a 2-3 fold increase in the amounts of DAG and TAG (Lee et al., 2017). On the other hand, the reduction in MGDG was interpreted to be due to induced breakdown due to ER stress rather than inhibition of its synthesis, thus releasing fatty acids which could then contribute to TAG synthesis (Lee et al., 2017). Therefore, the effects of downregulating BTA1 expression suggested that DGTS plays a significant role in the accumulation of TAG in *C. reinhardtii*.

Due to structural differences between DGTS and PC, the mechanism by which DGTS contributes to TAG accumulation likely differs from that of PC. DGTS contains an ether bond linking its headgroup to the glycerol backbone (**Figure 1**), making it unlikely for DGTS’s headgroup to be chemically or enzymatically removed to form DAG as occurs in PC. Enzymes capable of breaking this ether bond have not yet been identified, but are presumed to exist in order for cells to catabolize DGTS. Moreover, the vast majority of molecular species of DGTS contain a C18 fatty acid on the *sn*-2 position (Giroud et al., 1988) while ~90% of TAG molecular species contain a C16 fatty acid on the *sn*-2 position (Fan et al., 2011), thus supporting the idea that DGTS is not a direct precursor to DAG for TAG assembly. Therefore, fatty acid flux from DGTS into TAG probably occurs *via* the action of lipases or acyltransferases. In support of this hypothesis, Vogel and Eichenberger, 1992 observed turnover of radiolabel in the 18:2 fatty acid of DGTS that was substantially faster than the turnover of its polar headgroup. Similarly, ^13^C-labeling of *C. reinhardtii* lipids followed by an unlabeled chase into nitrogen deprivation revealed very little turnover of DGTS’s glycerol backbone and homoserine betaine headgroup compared to its fatty acids (Young et al., 2022). In addition, several studies have found that the quantity of DGTS stays constant in *C. reinhardtii* cells during nitrogen deprivation (Fan et al., 2011; Yang et al., 2018), despite high rates of TAG accumulation and the occurrence of fatty acid flux from DGTS into TAG. Therefore, it is probable that fatty acids from DGTS reach TAG by deacylation rather than removal of the betaine headgroup to form a DAG molecule that is then converted into TAG.

On the other hand, there is evidence that MGDG rather than DGTS is converted into DAG for TAG synthesis in *C. reinhardtii*. Lipidomic analysis of heat-stressed *C. reinhardtii* revealed a strong decrease in the major MGDG molecular species and an accumulation of this species in DAG and TAG (Légeret et al., 2016). This finding was corroborated by their use of a *crfad7* mutant, which altered the major MGDG molecular species and confirmed its decrease under heat stress and accumulation in DAG and TAG (Légeret et al., 2016). In accordance with this, Young et al., 2022 found evidence of MGDG recycling into DAG and TAG in *C. reinhardtii* during nitrogen deprivation, as isotopic label decreased in the major MGDG species and increased in the corresponding species of DAG and TAG. In addition, the stereochemical positions of the characteristic fatty acids of the major MGDG species were matched to the identical positions in DAG and TAG (Young et al., 2022). Data mining of the *C. reinhardtii* lipid body proteome (Moellering and Benning, 2010) led to the identification of a putative galactosyl hydrolase gene (CrGH), and insertional mutants in this gene have reduced TAG content (Gu et al., 2021). Thus, the route of acyl flux through PC *via* headgroup removal appears to be replaced in microalgae by removal of the galactosyl headgroup of MGDG rather than headgroup removal of DGTS.

DGTS appears to play a role in phosphorus-sparing in microalgae. Under optimal growth conditions, the green alga *Chlorella kessleri* does not contain DGTS, and contains PC as the dominant extraplastidic lipid. Within 48 hours of transfer to phosphorus-deficient conditions, this alga almost completely replaces the phospholipids PC and phosphatidylethanolamine (PE) with an equivalent amount of DGTS (Oishi et al., 2022). Interestingly, the fatty acid composition of DGTS was nearly identical to that of PC and PE in phosphorus-replete conditions (Oishi et al., 2022). *C. kessleri* was found to contain a homolog of the *C. reinhardtii* BTA1 gene, and the expression of this gene appears to be induced under phosphorus-deprived conditions in order to lower the alga’s phosphorus demand (Oishi et al., 2022). Similar findings were reported in the heterokont *Nannochloropsis oceanica*, which contains both DGTS and PC under nutrient replete growth conditions. During phosphorus deprivation, PC is reduced by 95% in *N. oceanica*, while DGTS levels strongly increased, becoming the most abundant membrane lipid in the cells (Meng et al., 2019). Upon resupply of phosphorus to the cells, PC levels increased and DGTS fell to pre-deprivation levels (Meng et al., 2019). This study also found that DGTS appeared to replace PC as the substrate for C18 fatty acid desaturation and acyl remodeling under phosphorus deprivation (Meng et al., 2019). Thus, levels of DGTS and PC are inversely correlated, and DGTS appears to substitute for PC under phosphorus-deprived conditions due to it being a non phosphorus-containing lipid.

DGTS may also aid microalgae in adaptation to lower temperatures. When the haptophyte microalga *Pavlova lutheri* was subjected to a low temperature, an increase in the relative amount of PUFAs and betaine lipid was observed (Tatsuzawa and Takizawa, 1995). At 15°C, the relative percentage of betaine lipid in *P. lutheri* increased four-fold compared to cells grown at 25°C (Tatsuzawa and Takizawa, 1995). In the heterokont *N. oceanica*, mutants lacking either DGTS synthesis (*bta1*) or PC synthesis were generated in order to determine which lipid plays a role in adaptation to lower temperatures (Murakami et al., 2018). Only the *bta1* mutant which lacked DGTS synthesis displayed significantly impaired cell growth at low temperatures, indicating that DGTS is required for maintaining optimal growth at low temperatures (Murakami et al., 2018). In both *P. lutheri* and *N. oceanica*, DGTS contains a high a proportion of 20:5 fatty acid, therefore it is postulated that enhanced DGTS under low temperatures aids in maintaining membrane fluidity by having a high PUFA content (Tatsuzawa and Takizawa, 1995; Murakami et al., 2018).

## Evidence for acyl editing substrates in plants and microalgae apart from PC and DGTS

**Figure 7:**
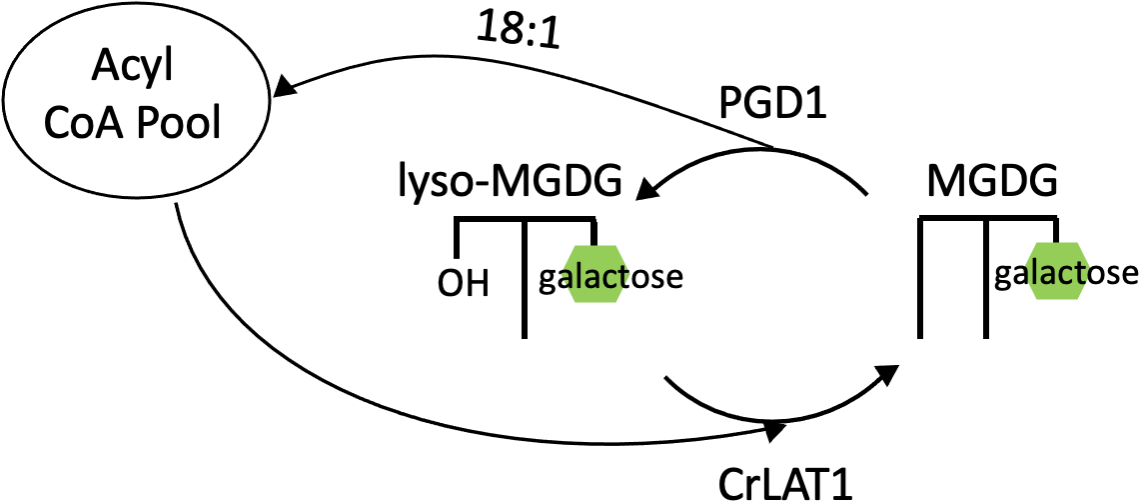
Acyl editing cycle of MGDG in *C. reinhardtii*. Removal of 18:1 from newly made MGDG at the *sn*-1 position by PGD1, and reacylation of lyso-MGDG catalyzed by CrLAT1. CrLAT1, *C. reinhardtii* Lysolipid Acyltransferase 1; MGDG, monogalactosyldiacylglycerol; PGD1, Plastid Galactoglycerolipid Degradation 1.

Indications of an MGDG acyl editing cycle in *C. reinhardtii* began with a study characterizing a galactoglycerolipid lipase gene named PLASTID GALACTOGLYCEROLIPID DEGRADATION1 (PGD1) (Li et al., 2012). This gene was discovered in an insertional mutant screen for lowered TAG content during nitrogen deprivation, and the insertion was found to be in a putative lipase-encoding gene. In terms of acyl composition, the *pgd1* mutant contained a lower amount of oleic acid (18:1) in TAG (Li et al., 2012). *In vitro* lipase assays revealed MGDG to be the substrate of the PGD1 lipase, and PGD1 preferentially hydrolyzes newly synthesized MGDG (18:1Δ9/16:0) at the *sn-1* position (Li et al., 2012). Pulse-chase analyses using ^14^C-acetate revealed that the proportion of label remained higher in MGDG and DGDG in the *pgd1* mutant during nitrogen deprivation, whereas in the wild type the label decreased in membrane lipids as it increased in TAG (Li et al., 2012). Thus, PGD1 is a lipase that preferentially releases 18:1Δ9 from newly-made MGDG, and the released fatty acid joins the acyl-CoA pool where it is available to contribute to TAG synthesis (**Figure 7**).

A follow up study further explored the role of PGD1 in membrane lipid turnover and remodeling in *C. reinhardtii* in response to unfavorable environmental conditions (Du et al., 2018). In this study, sensitivity of lipid analyses was increased by isolating chloroplasts, and an increased level of MGDG in *pgd1* mutants compared to wild type was observed (Du et al., 2018). In terms of MGDG acyl composition, a lower quantity of 16:3 and 18:2 was observed but a higher amount of 16:4 and 18:3 in *pgd1* cells compared to wild type (Du et al., 2018). This supports the model of PGD1-mediated turnover of MGDG 18:1Δ9 into TAG, as *pgd1* mutants are impaired in the flux of 18:1 from MGDG into TAG, thereby allowing oleate to reside in MGDG longer and thus become desaturated. Thus, these studies point to an acyl editing cycle on MGDG during nitrogen-deprived conditions.

Recently, an enzyme that appears to catalyze the reacylation of lyso-MGDG (MGDG with one fatty acid removed) has been characterized, and this activity would complete an acyl editing cycle of MGDG. A homolog of the *Arabidopsis thaliana* LPCAT1 gene was discovered in *C. reinhardtii* (CrLAT1), and this gene contained a conserved membrane-bound *O*-acyl transferase (MBOAT) domain (Iwai et al., 2021). Knockdown *CrLAT1* mutants contained an increased proportion of lyso-MGDG and decreased TAG accumulation compared to wild type cells (Iwai et al., 2021). In contrast, overexpression *CrLAT1* mutants contained a lower proportion of lyso-MGDG compared to wild type cells (Iwai et al., 2021). The most abundant fatty acid in lyso-MGDG was 16:4 with a minor proportion of other C16 fatty acids, supporting a model in which CrLAT1 acylates lyso-MGDG with a C18 fatty acid. This acyl editing mechanism in *C. reinhardtii* consists of PGD1 hydrolyzing newly-made 18:1Δ9/16:0 MGDG, releasing 18:1Δ9 to the cytosolic acyl-CoA pool where it is available for TAG synthesis, and CrLAT1 may catalyze the reacylation of lyso-MGDG at the *sn*-1 position with a C18 fatty acid (**Figure 7**).

Evidence of acyl editing on lipid substrates other than PC or DGTS has also been observed in land plants. In one study, a Δ6 desaturase from *Physcomitrella patens* was introduced into *Arabidopsis*, which lacks a Δ6 desaturase. The Δ6 desaturase is ER-localized with a preference for the *sn*-2 position of PC, and indeed in the transformant ~90% of the Δ6 fatty acids in PC were located in the *sn*-2 position (Hurlock et al., 2018). Δ6 acyl groups are synthesized in the ER and must be imported into the chloroplast, and they would presumably be located at the *sn*-2 position of chloroplastic lipids if acyl editing does not occur. However, in each of the chloroplastic lipids [MGDG, DGDG, sulfoquinovosyldiacylglycerol (SQDG), and phosphatidylglycerol (PG)], the Δ6 acyl groups were approximately equally distributed between the *sn*-1 and *sn*-2 positions, indicating that acyl editing of plastidial lipids does occur (Hurlock et al., 2018). Interestingly, PA isolated from whole leaf tissues displayed the same stereochemical distribution of Δ6 fatty acids as PC, while PA isolated from chloroplasts displayed the reverse stereochemistry with the majority of the Δ6 fatty acids located at the *sn*-1 position (Hurlock et al., 2018). Thus, some portion of PA is likely subject to acyl editing as well. Furthermore, through liquid chromatography mass spectrometry (LC-MS) a 34:7 MGDG molecular species was identified that contained one ER-derived fatty acid and one chloroplast-derived fatty acid, as it contained one 18:4 fatty acid with a Δ6 desaturation synthesized in the ER and one 16:3 fatty acid that is synthesized exclusively in chloroplast *via* the FAD5 desaturase (Hurlock et al., 2018). This supports the idea that acyl editing occurs on MGDG, as one exclusively ER-derived fatty acid and one exclusively chloroplast-derived fatty acid were found on the same molecule. However, the transgenic lines appeared to have higher lipid turnover compared to wild type (Hurlock et al., 2018), therefore it is possible that increased acyl editing may have been triggered in the transgenic line.

**Figure 8:**
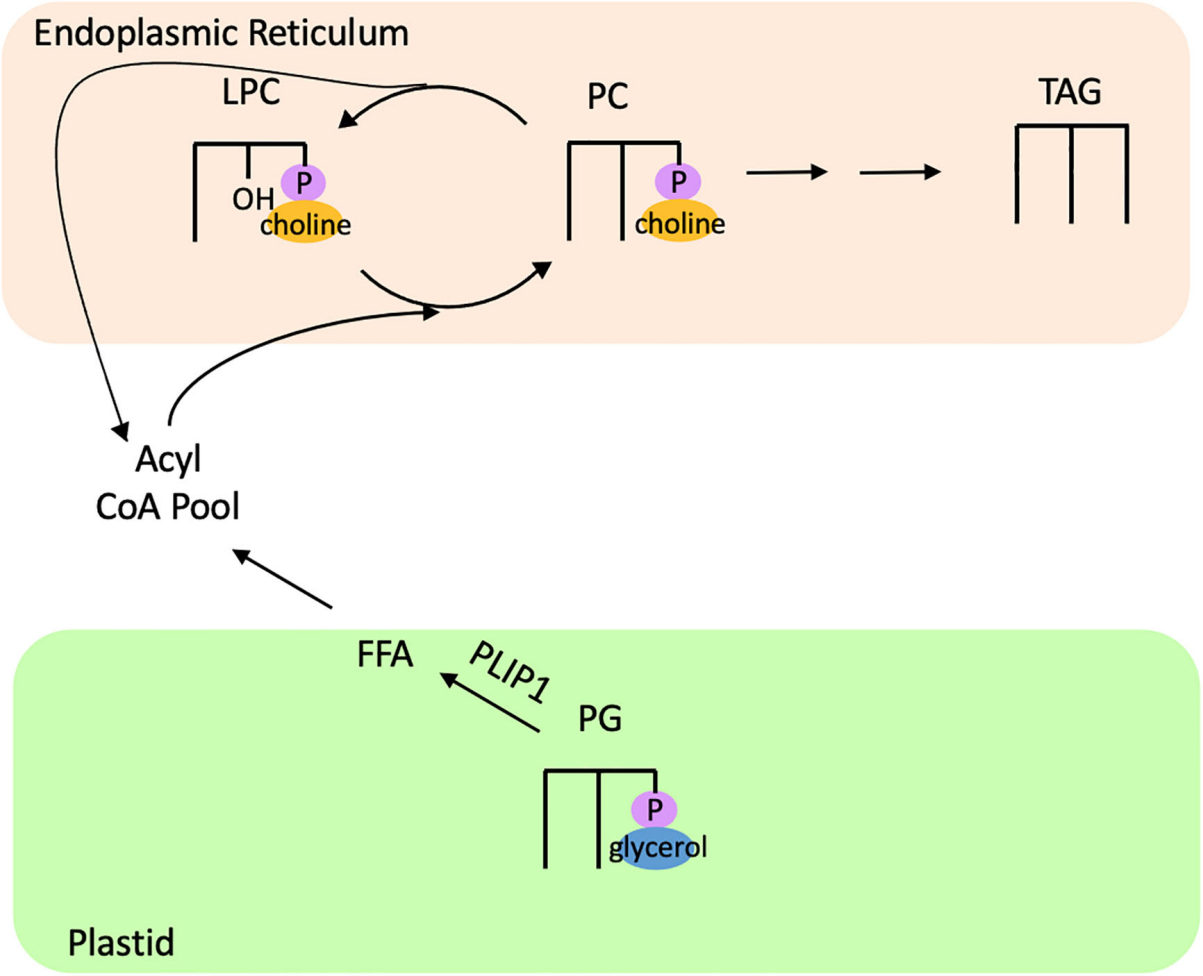
Acyl editing of PG in *Arabidopsis* contributes to TAG synthesis. The lipase PLIP1 releases 18:3 from 18:3/16:1Δ3t PG, which is channeled into PC and eventually incorporated into TAG. FFA, free fatty acid; LPC, lysophosphatidylcholine; PC, phosphatidylcholine; PG, phosphatidylglycerol; PLIP1, Plastid Lipase 1; TAG, triacylglycerol.

One study characterizing the function of a phospholipase A_1_, PLASTID LIPASE1 (PLIP1), in *Arabidopsis* found evidence of acyl editing on the plastidic phospholipid PG. PLIP1 contains a conserved Lipase 3 domain, similar to the PGD1 lipase of *C. reinhardtii* (Wang et al., 2017). *In vitro* assays of PLIP1 revealed that its preferred substrate is 18:3/16:1Δ3t PG, and that PLIP1 acts on the *sn-1* position to release the 18:3 fatty acid (Wang et al., 2018). A pulse-chase experiment using ^14^C-acetate in leaves of PLIP1 overexpressor lines in *Arabidopsis* revealed rapid incorporation of label in PG compared to wild type, with PG containing ~70% of the total label by the end of the pulse phase (Wang et al., 2018). During the chase phase, PG rapidly lost most of its label while PC rapidly accumulated label (Wang et al., 2017). A ^14^C-acetate pulse-chase experiment in developing *Arabidopsis* seeds also demonstrated higher incorporation of label into PG compared to wild type seeds and increased turnover of PG during the chase period, while label concomitantly turned over in PC and accumulated heavily in TAG (Wang et al., 2018). Furthermore, *plip1* insertional mutants displayed ~10% lowered seed fatty acid content (the majority of which is in TAG) compared to wild type, while PLIP1 overexpressors had a 40-50% increase in seed fatty acid content (Wang et al., 2018). Taken together, these results indicate that PLIP1 transfers a polyunsaturated fatty acid from PG into PC, and this fatty acid fluxes through PC and contributes to TAG synthesis (**Figure 8**). Thus, PLIP1 appears to be part of an acyl editing cycle that acts on PG and contributes to the flux of PUFAs through PC into TAG.

In addition, the lipase HEAT INDUCIBLE LIPASE1 (HIL1) in *Arabidopsis* was characterized *via in vitro* lipase activity assays and found to have a substrate preference for MGDG (Higashi et al., 2018). Insertional *hil1* mutants are impaired in the remodeling of 18:3 into TAG during heat stress (Higashi et al., 2018), thus indicating that HIL1 plays a role in the turnover of PUFAs from MGDG to TAG.

Previous studies demonstrated that LPCAT can catalyze both the acylation and deacylation of PC *via* the forward and reverse reactions, respectively (Lager et al., 2013). *In vitro* assays investigated the capability of *Arabidopsis* LPCAT2 and *Arabidopsis* lysophosphatidylethanolamine acyltransferase (LPEAT2) to catalyze the forward and reverse reactions in the phospholipids PE and PA (Jasieniecka-Gazarkiewicz et al., 2016). This study found that although both AtLPCAT2 and AtLPEAT2 could catalyze the reverse reaction (deacylating an intact phospholipid), the activity of the reverse reaction varied greatly between phospholipids and was the lowest for PA (Jasieniecka-Gazarkiewicz et al., 2016). However, these enzymatic assays established the possibility of acyl remodeling of PE and PA. The substrate specificity and forward and reverse activity of LPAAT and LPEAT were then assayed in microsomal fractions of *Camelina sativa* seeds (Klińska et al., 2020). In terms of substrate specificity in the forward reaction (i.e. acylating a lyso-phospholipid to form a phospholipid), 16:0 and unsaturated C18 fatty acids were the preferred substrates of LPAAT, while unsaturated C18 fatty acids were the preferred acyl donors in the LPEAT-catalyzed reaction (Klińska et al., 2020). The activity of the reverse reaction (deacylating a phospholipid) was assayed by incubating ^14^C-acyl-CoAs with PA or PE, and it was found that both LPAAT and LPEAT could catalyze the backward reaction, although the acyl donor used affected the degree of activity observed (Klińska et al., 2020). Although the back-reaction could be performed by LPEAT, PE displayed a very slow remodeling rate and only contributed about 2% to the fatty acids in mature *C. sativa* seeds (in which TAG comprises ~93% of total lipids), despite PE constituting ~13-20% of all polar lipids (Pollard et al., 2015; Klińska et al., 2020). However, PE may donate a fatty acid to TAG synthesis *via* the action of PDAT, and the resulting lyso-PE could be reacylated with acyl-CoA by LPEAT. Interestingly, although PA only constitutes a minor fraction of polar lipids in *C. sativa* seeds (~2-4%), it appears ~5% of fatty acids in mature *C. sativa* seeds are first esterified to PA, before being transferred to the acyl-CoA pool via LPAATs (Klińska et al., 2020). This degree of acyl editing on PA is surprising given that desaturation is not known to occur on PA, although PA constitutes a relatively small proportion of membrane lipids and ultimately is not a substantial fatty acid contributor to seed oil content. Thus, PA and PE are potential substrates of acyl remodeling in plants, although neither seems to contribute flux as significant as PC.

## Discussion

**Figure 9:**
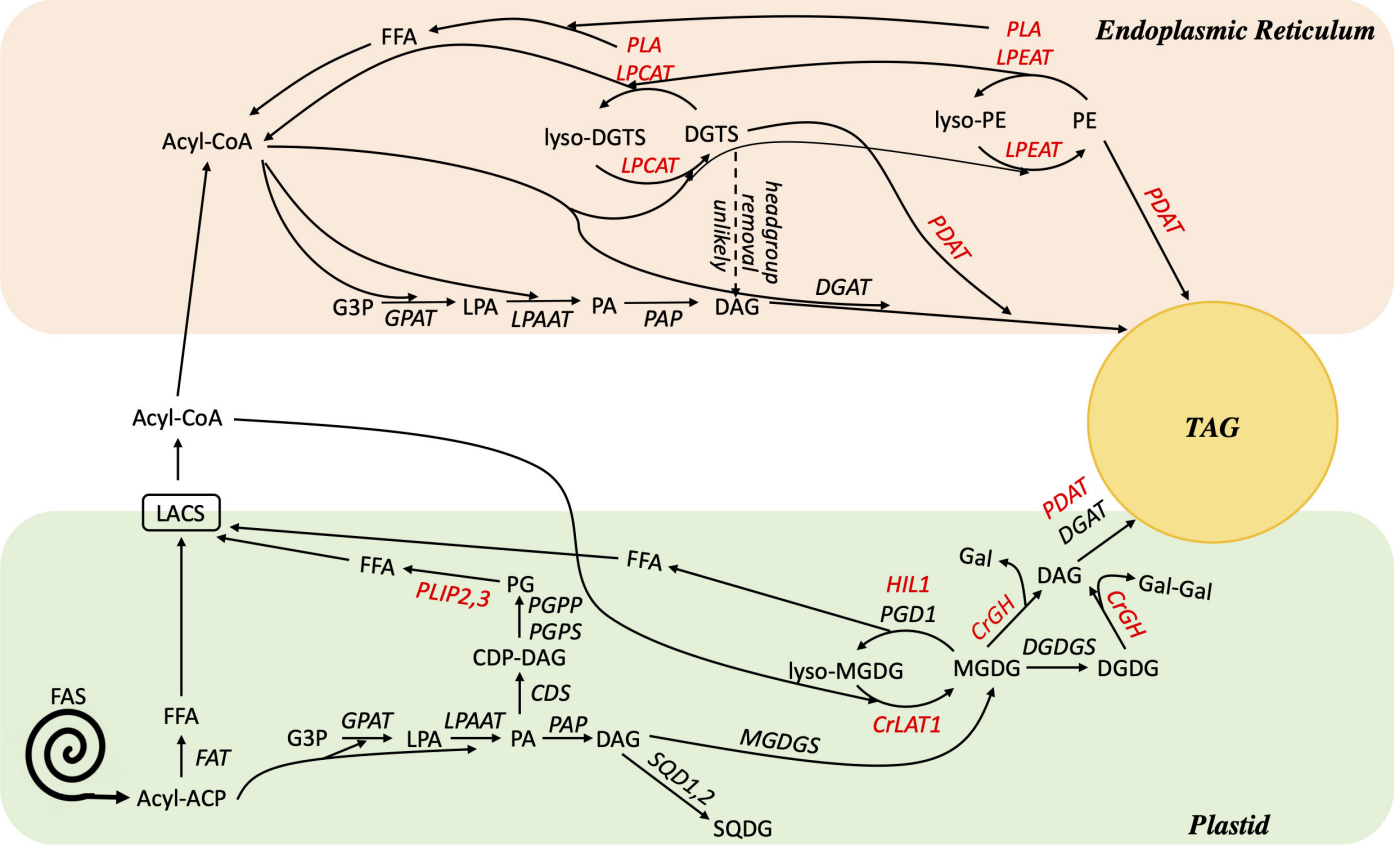
Working model of lipid metabolism in *C. reinhardtii* during TAG accumulation. Enzymes are shown in italics, for those shown in red further evidence is needed to test their role(s) and/or substrates *in vivo*. A dashed arrow indicates that for DGTS, headgroup removal or exchange is unlikely. Biosynthesis reactions of extraplastidial lipids DGTS, PE, and PI are omitted for simplicity. ACP, acyl carrier protein; CDP-DAG, cytidine diphosphate diacylglycerol; CDS, CDP-DAG synthase, CrGH, *C. reinhardtii* galactosyl hydrolase; CrLAT1, *C. reinhardtii* Lysolipid Acyltransferase 1; DAG, diacylglycerol; DGAT, diacylglycerol acyltransferase; DGDGS, digalactoslydiacylglycerol synthase; DGTS, diacylglyceryl-N,N,N-trimethylhomoserine; FAS, fatty acid synthase; FAT, fatty acyl-ACP thioesterase; FFA, free fatty acid; G3P, glycerol 3-phosphate; Gal, galactose; GPAT, glycerol-3-phosphate acyltransferase; HIL1, heat inducible lipase 1; LACS, long chain acyl-CoA synthetase; LPA, lysophosphatidic acid; LPAAT, lysophosphatidic acid acyltransferase; LPCAT, acyl-CoA:lysophosphatidylcholine acyltransferase; LPEAT, lysophosphatidylethanolamine acyltransferase; MGDG, monogalactosyldiacylglycerol; MGDGS, monogalactosyldiacylglycerol synthase; PA, phosphatidic acid; PAP, phosphatidic acid phosphatase; PDAT, phospholipid:diacylglycerol acyltransferase; PE, phosphatidylethanolamine; PG, phosphatidylglycerol; PGD1, Plastid Galactoglycerolipid Degradation 1; PGPP, phosphatidylglycerol phosphate phosphatase; PGPS, phosphatidylglycerolphosphate synthase; PLA, phospholipase; PLIP2, 3, plastid lipase 2, 3; SQD1, 2, sulfoquinovosyldiacylglycerol synthase; SQDG, sulfoquinovosyldiacylglycerol; TAG, triacylglycerol.

Acyl flux through PC serves several important functions in plants, including acting as a major site of extraplastidial desaturation, as a distributor of PUFAs into other lipids *via* acyl editing, and as an important hub of acyl flux into the neutral lipid TAG. Interestingly, the model green microalga *C. reinhardtii* completely lacks PC, and many species of microalgae contain highly reduced PC content, thus raising the question of if and how the functions of PC are replaced in microalgae. Given their inverse relationship and the structural similarity of DGTS and other betaine lipids to PC, it seems reasonable to postulate that betaine lipids substitute for the functions of PC in microalgae. Consistent with such roles is the evidence described above of rapid incorporation of exogenous fatty acids into DGTS, extraplastidial desaturation on DGTS, and substantial fatty acid flux through DGTS into TAG. However, the structure of DGTS makes it highly improbable that an analogous biochemical reaction of PC headgroup removal could occur on DGTS, and studies have found evidence of slower turnover of the DGTS backbone and headgroup compared to its fatty acids (Vogel and Eichenberger, 1992; Young et al., 2022). Additionally, knockout and characterization of the *C. reinhardtii* homolog of PDAT have not shown that DGTS replaces PC as the substrate of this enzyme in algae (Boyle et al., 2012; Yoon et al., 2012). In fact, phylogenetic analysis of the PDAT protein revealed the divergent evolution of PDAT in plants and algae and suggested that PDAT has a diminished and/or different role in green algae (Falarz et al., 2020). Similarly, knockdown of the *C. reinhardtii* homolog of *Arabidopsis* LPCAT revealed altered turnover of MGDG rather than DGTS (Iwai et al., 2021). Taken together, it seems likely that DGTS takes on some, but not all, of the roles in algae that PC has in plants. This is illustrated in **Figure 9**, which highlights known and potential pathways of fatty acid editing in algae.

In addition to the functions described above, PC may also play a role in lipid trafficking. The eukaryotic pathway of galactolipid synthesis requires transport of some lipid precursor from the ER to the plastid. The *Arabidopsis* trigalactosyldiacylglycerol (TGD) transmembrane protein complex of the chloroplast has been shown to conduct ER-to-plastid lipid trafficking (Xu et al., 2003; Xu et al., 2010; Fan et al., 2015), although its transported lipid substrate remains unknown. Potential candidates for the transported lipid substrate include PC, lyso-PC, DAG, or PA, but regardless of its identity the transported lipid is believed to be derived from PC. Interestingly, recent studies suggest that the PC involved in transport to the chloroplast for galactolipid synthesis is metabolically distinct from the PC pool utilized for acyl editing (Karki et al., 2019). Intriguingly, despite its lack of PC *C. reinhardtii* contains an ortholog of the plant TGD transporter called CrTGD2, and isotopic labeling experiments indicated that *Chlamydomonas* can import lipid precursors from the ER (Warakanont et al., 2015). It is postulated that PA may be the lipid species transported from the ER to the chloroplast in *C. reinhardtii*. It remains to be determined what role DGTS may have in lipid trafficking in algae.

Thus, the extent to which betaine or other lipids replace the roles of PC in algae is an area ripe for future study. While radiolabeling has been instrumental in confirming some of the functions of DGTS, more detailed ^13^C isotopic labeling time courses should be performed in order to trace the fatty acid fluxes of DGTS in algae. The relative amount of fatty acid modification and export from DGTS into other lipids such as TAG should be estimated by ^13^C and/or other isotopic labeling to determine its potential contribution as an acyl hub. Prior ^13^C-labeling studies have demonstrated that DGTS contributes substantial fatty acid flux toward TAG synthesis (Young and Shachar-Hill, 2021), and future studies examining the turnover of individual lipid components on a finer timescale can be expected to illuminate the mechanism of DGTS’s contribution to the synthesis of TAG and other glycerolipids. In addition, we believe that more detailed analysis of algal homologs of plant enzymes known to conduct acyl flux through PC including LPCAT, PDAT, and phospholipase A2 would be valuable. Such analyses should include *in vitro* enzymatic assays to ascertain whether DGTS may replace PC as the preferred substrate of these enzymes. Finally, the use of [^13^C_2_^18^O_2_]acetate labeling may shed light onto the role of DGTS and other lipids as acyl editing hubs, as ^18^O fatty acid labeling can help determine the number of hydrolysis reactions that have taken place, thus informing the history of a fatty acid and possible routes of its trafficking (Pollard and Ohlrogge, 1999). Given the numerous critical functions of PC in plant lipid metabolism, determining the extent to which betaine or other lipids replace the function of PC in algae is important for understanding and engineering algal lipid metabolism.

## Author contributions

DH and YS-H discussed and conceived of the ideas in this paper. DH researched, wrote the article, and prepared the figures. YS-H contributed to the final form of the manuscript. All authors contributed to the article and approved the submitted version.

## Glossary

**Table:**

BTA1	Betaine Lipid Synthase 1
CPT	CDP-choline:1 2-Diacylglycerol Cholinephosphotransferase
CrLAT1	C. reinhardtii Lysolipid Acyltransferase 1
DAG	Diacylglycerol;
DGAT	Di a c y l g lyc e ro l Acy l t ransfer a se
DGDG	Diga lactosy l d iacyl g lycerol
DGTA	Diacylglycerylhydroxymethyltrimethyl-b-Alanine
DGTS	Diacylglyceryl-N
N	N-trimethylhomoserine
ER	Endoplasmic Reticulum
FA	Fatty Acid
FFA	Free Fatty Acid
FAD	Fatty Acid Desaturase
G3P	Glycerol 3-Phosphate
GPAT	Glycerol-3-Phosphate Acyltransferase
GPC	Glycerolphosphocholine
GPCAT	Glycerolphosphocholine Acyltransferase
LC-MS	Liquid Chromatography Mass Spectrometry
LPAAT	Lysophosphatidic Acid Acyltransferase
LPCAT	Lysophosphatidylcholine Acyltransferase
LPCT	Lysophosphatidylcholine Transacylase
LPEAT	Lysophosphatidylethanolamine Acyltransferase
LPLAT	Lysophospholipid Acyltransferase
MGDG	Monogalactosyldiacylglycerol
PA	Phosphatidic Acid
PC	Phosphatidylcholine
PDAT	Phospholipid : Diacylglycerol Acyltransferase
PDCT	Phosphatidylcholine : Diacylglycerol Cholinephosphotransferase
PE	Phosphatidylethanolamine
PG	Phosphatidylglycerol
PGD1	Plastid Galactoglycerolipid Degradation 1
PLIP1	Plastid Lipase 1
PUFA	Polyunsaturated Fatty Acid
SQDG	Sulfoquinovosyldiacylglycerol
TAG	Triacylglycerol
TGD	Trigalactosyldiacylglycerol
